# Effects of Sudden Stoppage of Hypodermic Morphia

**Published:** 1879-06-20

**Authors:** 


### EFFECTS OF SUDDEN STOPPAGE OF HYPODERMIC MORPHIA.

Jas. Braithwaite, M.D. (London Lancet), gives a case where a lady who, for seven years, had taken morphia hypodermically—the dose at one time amounting to fourteen grains a day—suddenly discontinued its use. He says :

Just a forthnight after her confinement, at her own urgent request, and with the concurrence of Mr. Jessop, it was decided that the morphia should be discontinued at once and entirely. She was injected for the last time on the morning of February 20th. We had no facts as to other cases to guide us, except a short note of a somewhat similar case published in an old number of the Edinburgh Medical Journal, although not with reference to the use of morphia hypodermically. This, however, as it turned out, led us considerably to under-estimate the seriousness of the illness which would follow. Constant, and indeed incessant vomiting and purging came on next day. The vomiting occurred about every ten minutes, and consisted of a mucoid fluid tinged with bile. The alvine evacuations were similar in appearance, but darker and more tenacious.

On the second day the vomiting and purging still continued, as they had done all night, the stomach rejecting everything, and even a nutrient enema by the bowel coming away at once. Ice, ice and champagne, milk, and lime-water, in very small quantities, were tried in vain.

On the fifth morning the stomach retained for a short time a morsel of chicken, with a particle of bread, and about a dessertspoonful of beer; but this was rejected in half an hour, and appeared to have aggravated the sickness. The stomach retained no food for more than a few minutes, until on the ninth day her life appeared in great danger—so great that I had the syringe ready charged, and urged her to let me inject her to save her life. She, however, was firm in her resolution, and said she would rather die than again become the slave of the morphia.

The next day (the tenth) the stomach retained food (a morsel of fish) for a few hours, and after this the improvement was gradual, but certain. The diarrhœa, however, continued more or less, and the stomach would only bear food on one condition—that a very long interval should elapse between each meal.

I believe the case would have ended here, but through some over-exertion the erysipelas returned on April 16th, and rapidly spread. This seemed to cause a renewal of all the old sickness and diarrhœa, and this lasted continuously for ten days more distressingly than before, and accompanied with such burning of the throat and mouth as to oblige her to have a wet sponge laid upon her lips.

After the tenth day improvement gradually, but much more slowly than on the previous occasion, set in; but up to this date (December, 1878) the diarrhœa still remains, and on the least over-exertion becomes aggravated. My impression is that it might have been got rid of long before this by absolute quiet and rest, but unfortunately I have never been able to enforce this, and the diarrhœa has now become so chronic that rest has very little effect upon it. There can be no question, however, that every fresh exertion causes an exacerbation of it.

I conclude from this case that a very gradual diminution of morphia injected is preferable to a sudden discontinuance of it; and seeing that in two years she has got down from fourteen grains a day to three quarters of a grain, a gradual diminution is proved perfectly practicable. It is, however, the opinion of this lady that there is a greater totality of suffering, but spread, of course, over a longer period of time. If, however, from any reason, (as formation of abscess) it is discontinued suddenly, the symptoms resulting will be incessant vomiting and diarrhœa. The vomiting will subside in a variable period, and the sooner the less the stomach is teased with anything. The diarrhœa, however, will continue for a long period, and is best treated by absolute bodily repose. Neither astringents nor enemata of any kind have any effect in checking it; rather the contrary. An enema of thirty grains of chloral hydrate in an ounce of tepid water at bedtime must be excepted, as this was retained, and proved of service on several occasions.—*London Med. News.*

